# Incidence and risk factors of *Blastocystis *infection in an orphanage in Bangkok, Thailand

**DOI:** 10.1186/1756-3305-5-37

**Published:** 2012-02-14

**Authors:** Duangnate Pipatsatitpong, Ram Rangsin, Saovanee Leelayoova, Tawee Naaglor, Mathirut Mungthin

**Affiliations:** 1 Biomedical Sciences Program, Faculty of Allied Health Sciences, Thammasat University, Khlong Luang, Pathum Thani 12121, Thailand; 2 Department of Military and Community Medicine, Phramongkutklao College of Medicine, Ratchathewi, Bangkok 10400, Thailand; 3 Department of Parasitology, Phramongkutklao College of Medicine, Ratchathewi, Bangkok 10400, Thailand

## Abstract

**Background:**

*Blastocystis *sp. is one of the most common intestinal protozoa in humans. Unlike other intestinal parasitic infections such as giardiasis and cryptosporidiosis, the epidemiology of blastocystosis in children who live in crowded settings such as day-care centers and orphanages has been rarely explored.

**Methods:**

A retrospective cohort study was conducted to evaluate incidence and risk factors of *Blastocystis *infection in an orphanage every two consecutive months during April 2003 to April 2004, in Bangkok, Thailand. *Blastocystis *sp. was identified using direct simple smear, and *in vitro *cultivation in Jones' medium.

**Results:**

The incidence rate was 1.8/100 person-months and the independent risk factors associated with *Blastocystis *infection were age, nutritional status and orphans living in the room where their childcare workers were infected.

**Conclusions:**

Person-to-person transmission was most likely to occur either from orphans to childcare workers or from childcare workers to orphans living in the same room. Universal precautions such as regular hand washing and careful handling of fecally contaminated materials are indicated.

## Background

*Blastocystis *sp., one of the most common intestinal protozoa in humans, has a worldwide distribution and is frequently seen in stool samples identified in parasitological surveys. *Blastocystis *sp. had been thought to be a commensal organism since most cases of blastocystosis have been reported as asymptomatic carriages [1-3]. However, a few studies have identified *Blastocystis *sp. as a causative agent of gastrointestinal diseases [4-6]. The high prevalence of 10-40% of *Blastocystis *sp. has been reported in developing countries [7,8] while in Thailand, the prevalence of *Blastocystis *carriage in various groups is as high as 11-37% [9-11]. Risk factors for acquiring blastocystosis have been identified, and include contact with animals and consuming contaminated food or water [12-14]. Most studies of *Blastocystis *infection in young Thai children were performed using cross-sectional design [15-18] so the epidemiological information including incidence, true risk factors and natural history of this infection is still lacking. In addition, compared to other intestinal parasitic infections such as giardiasis and cryptosporidiosis, the epidemiology of blastocystosis in children who live in crowded settings such as day-care centers and orphanages has been rarely explored. Thus, the objectives of this study were to determine the incidence of and risk factors for *Blastocystis *infection in an orphanage, Bangkok, Thailand.

## Methods

### Study population

A retrospective cohort study was conducted in Thai orphans who lived in an orphanage located in Bangkok, Thailand from April 2003 to April 2004. This orphanage is for orphans aged less than five years and consisted of 10 rooms for orphans, accommodating 30 to 40 orphans with 3 childcare workers and a food and milk preparation room. The orphans were raised in the institute until they were adopted. Otherwise, they were transferred to other orphanages for older children at the age of five. These children were rarely brought out of the area. The childcare workers in each room were asked to collect stool samples and complete standardized questionnaires for these orphans for whom they were responsible every two consecutive months. The study was reviewed and approved by the Ethical Committee of the Medical Department, the Royal Thai Army.

### Stool collection and examination

A total of 697 fecal samples were collected and transported to the Department of Parasitology, Phramongkutklao College of Medicine, Bangkok, Thailand. To identify *Blastocystis *sp., each stool sample was screened by direct simple smear. Stool samples were then cultured in Jones medium supplemented with 10% horse serum. After incubation for 48 to 72 h at 37°C, each sample was examined by light microscopy. A *Blastocystis*-infected case was defined as the presence of vacuolar, cyst, trophozoite or other forms in the culture of stool specimens.

### Questionnaire

To determine the risk factors and outcomes of *Blastocystis *infection, standardized questionnaires were collected regarding age, sex, weight, height, underlying disease, HIV infection status, and present illnesses including diarrhea, abdominal pain and other gastrointestinal symptoms. Diarrhea was defined as a change in their normal pattern of bowel movements and at least three loose stools during a 24-hour period. Dysentery was defined as at least one passage of mucous bloody stool in one day. The weight and the height of individuals were recorded at the nursing room. To determine their nutritional status, criteria from the Institute of Nutrition, Mahidol University (INMU) Thai Growth Study was used [19].

### Statistical analysis

Incidence was defined as the number of new cases occurring during the observation period. The estimated date of infection for the incident cases was taken as the midpoint between the last negative result and first positive result. Survival analysis was used to calculate median time to clearance of *Blastocystis *infection. The estimated date of the time to clearance was taken from the period between the first positive results to the first negative results of *Blastocystis *identification. Difference between the median times to clearance was analyzed by Log-rank test. Possible risk factors were analyzed using incidence rate ratios and their 95% confidence intervals. The chi-square or Fisher's exact test was used to compare proportions. Poisson regression using STATA/SE for Window version 9.2 (StataCorp LP, College Station, TX) was performed for multivariate analysis to assess the independent association of the risk factors and *Blastocystis *infection.

## Results

### Study population

A total of 697 participants, composed of 606 orphans and 91 childcare workers, were enrolled in the study. The median ages of the orphans and childcare workers were 10.5 months (0.3 months to 9.0 years) and 37.0 years (20.0 to 52.0 years), respectively. The number of participants who enrolled every two consecutive months was 343, 336, 336, 319, 287, 339 and 305, respectively (Table 1). Ninety-eight orphans (14.1%) were HIV positive (57 males and 41 females). Information on CD4^+ ^T-lymphocyte count was not available. All HIV-positive orphans were prescribed antiretroviral therapy (i.e., zidovudine and didanosine).

**Table 1:** Prevalence, incidence and incidence rate of *Blastocystis *infection in the orphanage, Bangkok, Thailand from April 2003 to April 2004.

	Prevalence	Incidence	Time	Incidence rate	Total
	N (%)	N (%)	(person- months)	(100 person- months)	
April 2003	94 (27.4)	-	-	-	343
June 2003	35 (10.4)	9 (2.7)	450.2	1.9	336
August 2003	29 (8.6)	9 (2.7)	470.9	1.9	336
October 2003	23 (7.2)	5 (1.6)	259.1	1.9	319
December 2003	19 (6.6)	9 (3.1)	283.3	3.2	287
February 2004	26 (7.7)	6 (1.8)	462.6	1.3	339
April 2004	26 (8.5)	8 (2.6)	433.1	1.8	305

### Characterization of persons with *Blastocystis *infection

The characteristics of the enrolled participants are shown in Table 2. *Blastocystis *infection was significantly different when analyzed by age group (*p *< 0.001), rooms (*p *< 0.001) and reception of care from an infected childcare worker. (*p *= 0.002). No significant difference was found between sex, HIV infection, nutritional status and diarrhea. In addition, no other symptoms including abdominal pain, flatulence, anorexia and nausea/vomiting were associated with *Blastocystis *infection (*p *= 1, 0.150, 0.233 and 0.610, respectively).

**Table 2:** Characteristics of the enrolled subjects and the incidence of *Blastocystis *infection from April 2003 to April 2004.

Characteristics	No. of enrolled subjects	No. (%) infected	*p value *
Sex			
Male	232	22 (9.5)	
Female	182	15 (8.2)	0.660

Age (months)			
0-12	202	1 (0.5)	
13-24	93	3 (3.2)	
25-36	24	7 (29.2)	
37-48	26	6 (23.1)	
49-60	12	7 (58.3)	
> 60	56	13 (23.2)	< 0.001

Room no. (specific group)			
No. 1 (36-60 months)	32	15 (46.9)	
No. 2 (new born to 8 months)	47	1 (2.1)	
No. 3 (new enrolled)	67	0 (0.0)	
No. 4 (HIV- infected children)	69	9 (13.0)	
No. 5 (32-36 months)	21	5 (23.8)	
No. 6 (24-32 months)	22	3 (13.6)	
No. 7 (new born to 8 months)	37	1 (2.7)	
No. 8 (8-12 months)	42	1 (2.4)	
No. 9 (12-18 months)	50	1 (2.0)	
No. 10 (18-24 months)	23	0 (0.0)	
Food and milk preparation room	4	1 (25.0)	< 0.001

Nutritional status			
Normal nutrition	281	29 (10.3)	
Undernutrition	86	4 (4.7)	
Overnutrition	33	3 (9.1)	0.275

HIV infection			
No	330	27 (8.2)	
Yes	67	9 (13.4)	0.172

Infected childcare worker(s) in the room			
No	257	14 (5.4)	
Yes	119	18 (15.1)	0.002

Diarrhea			
No	371	31 (8.4)	
Yes	7	1 (14.3)	0.464

### Prevalence and incidence of *Blastocystis *infection

From a total of 697 participants, the prevalences, incidences and the incidence rates of *Blastocystis *carriage during each round are shown in Table 1. A total of 414 participants who were negative for *Blastocystis *infection at the baseline survey in April 2003 were enrolled and followed up every two consecutive months until April 2004. Of these *Blastocystis*-negative participants, 232 (56.0%) were male and 182 (44.0%) were female. The incidence rate of blastocystosis was 1.8/100 persons-months.

### Time to clearance of *Blastocystis *infection

Analysis of time to clearance was performed in a total of 115 infected participants whose first positive and first negative results were identified. Of these, 89 and 26 were children and childcare workers, respectively. The median time to clearance of *Blastocystis *infection in this population was 2.62 months (95%CI, 2.57-2.67). Subgroup analysis showed that the median time to clearance was 2.59 months (95%CI, 2.55-2.63) in children and 10.03 months (95%CI, 5.56-14.51) in childcare workers. Significant difference was found between the median times to clearance of these 2 groups with the *p *value of < 0.001.

### Risk factors of *Blastocystis *infection

Univariate analysis showed an increasing risk of acquisition of *Blastocystis *infection with age (IRR = 1.04, 95%CI, 1.03-1.05). Additionally, children who lived in rooms where childcare workers were infected with *Blastocystis *sp. had a higher risk of acquiring blastocystosis (IRR = 3.3, 95%CI, 1.7-6.7). Multivariate Poisson regression analysis showed that age was independently associated with *Blastocystis *infection. The risk of acquiring *Blastocystis *infection increased at a rate of 1.04 for each additional year of the subject's age. However, classification of age into 3 groups (< 2 yr, 2-5 yr, > 5 yr) showed that those who were 2 to 5 and greater than 5 years had higher risk of *Blastocystis *infection than those who were less than 2 years (*p *< 0.001, data not shown). Since each room was assigned for orphans within a specific age group, rooms were not included into the multivariate analysis model. Nutritional status showed that orphans who had undernutrition status had lower risk of infection than those with normal or overnutrition status. Additionally, children who lived in a room where childcare workers were also infected with *Blastocystis *sp. had a 3.1 times higher risk of acquiring *Blastocystis *infection (95%CI, 1.3-7.6) than those who lived in the rooms without infected childcare workers after adjusting for sex and HIV infection status. In contrast, sex and HIV status were not associated with blastocystosis (Table 3).

**Table 3:** Univariate and multivariate analysis for risk factors of *Blastocystis *infection.

Characteristics	No. positive for *Blastocystis*	Person-months of follow up	IR (100 person-months)	IRR (95% CI)			
				
				Crude	*p value *	Adjusted	*p value *
Sex							
Male	29	1491.1	1.9	1		1	
Female	17	1128.9	1.5	0.8 (0.4-1.5)	0.408	0.7 (0.3-1.5)	0.366
Age (months)	46	2620.0	1.8	1.04 (1.03-1.05)	< 0.001	1.04 (1.03-1.05)	< 0.001
Nutritional status							
Normal nutrition	37	1653.9	2.2	1		1	
Undernutrition	4	655.8	0.6	0.3 (0.1-0.8)	0.006	0.3 (0.1-0.9)	0.044
Overnutrition	4	215.9	1.9	0.8 (0.2-2.3)	0.851	0.5 (0.1-2.5)	0.417
HIV infection							
No	34	2116.9	1.6	1		1	
Yes	10	444.4	2.3	1.4 (0.6-2.9)	0.352	1.7 (0.6-4.9)	0.313

Infected childcare worker(s) in the room							
No	16	1613.8	0.9	1		1	
Yes	24	723.4	3.3	3.3 (1.7-6.7)	0.002	3.1 (1.3-7.6)	0.014

Data were adjusted for sex and HIV infection status. IR = incidence rate; IRR = incidence rate ratio

## Discussion

In this study, *Blastocystis *sp. was identified using both direct wet smears and stool culture. Short term *in vitro *cultivation is considered to be a diagnostic method with a high sensitivity for the detection of *Blastocystis *sp. [20]. The incidence rate of *Blastocystis *infection was 1.8/100 person-months in this setting, which was low compared with a previous study in children at an older age group [21]. The prevalences of *Blastocystis *carriage in orphans at each time point varied from 7.2% to 27.4%. The highest prevalence was shown at the beginning of the study, and then gradually decreased after the time of follow up. This might be due to the notification of the first finding. Additional interventions that occurred during early 2004 to prevent transmission of parasitological infections included health education and the cleaning of clothes and bedding accessories using autoclave heat treatment. Since *Blastocystis *is not a host-specific organism, transmission from human-to-human and animal-to-human could have occurred [22,23]. It is indicated that the orphans acquired *Blastocystis *infection in this orphanage since no newly-enrolled orphans were infected by *Blastocystis *at the enrollment. In this orphanage, zoonotic transmission was unlikely to have occurred since no pets were allowed to be raised in this community setting. Water contamination has been identified as a method of transmission [16,18,24]. In this orphanage, filtered water was supplied from the same source to be used as drinking water in every room. Because significant differences of *Blastocystis *infection were found among different rooms, it was less likely that water was the main route of transmission. In this study, the prevalence and incidence of blastocystosis was significantly different among rooms. Thus waterborne transmission of *Blastocystis *infection was less likely since drinking water for orphans was from the same source.

Univariate and multivariate analysis showed that older orphans had a higher risk of acquiring the infection than those at younger ages. This independent risk factor was probably related to the children's behaviors such as playing with each other on unclean floor areas or playground, poor toilet training and poor hygienic practices, e.g., food-handling. In contrast, younger children had been taken care of by their childcare workers. These results were similar to those reported in several studies, where *Blastocystis *sp. was often identified in older children and adults [9,15,25]. Most infected children in this orphanage had normal nutritional status. Analysis of risk associations showed that children who had undernutritional status had a significantly lower risk for acquiring the infection. From personal communication, these undernourished children were usually among the new enrollments. After living in the orphanage, they had received better care, and, thus, were less likely to be undernourished. This may indicate that acquiring blastocystosis was related to the period of time that they stayed in this orphanage.

The possibility of person-to-person transmission was shown when the association of infected orphans and their childcare workers was analyzed. A significant association was found between the infected childcare workers and the orphans under their responsibility. Orphans who lived in the room where childcare workers were infected with *Blastocystis *sp. had a higher risk of acquiring blastocystosis than those who lived in the rooms without infected child care workers. This evidence suggests person-to-person transmission of *Blastocystis *sp. in this community although no subtyping or phylogenetic analysis was performed to corroborate this finding. Survival analysis showed that the median time to clearance of *Blastocystis *infection in the childcare workers was 10.03 months. Suresh et al. (2009) studied *in vivo *encystation of *Blastocystis *sp. and found that *Blastocystis *survived in infected individuals over a 6-month period [26]. Zhou and colleagues (2010) inoculated *Blastocysti*s sp. into the abdominal cavity of mice, and found that only the cyst form existed for more than 6 months [27]. Both studies showed that encystations enable *Blastocystis *sp. to survive and avoid an immune attack from the host. The time to clearance of *Blastocystis *infection in the childcare workers was similar to those found in these studies. Therefore, it is likely that the infected childcare workers could consistently transmit *Blastocystis *sp. for a period of time. However, the median time to clearance of *Blastocystis *infection in the orphans was significantly shorter. This finding may reflect the recurrent exposure to *Blastocystis *in the childcare workers since they lived outside the orphanage. Further studies should be performed to identify the factors contributing to the difference in the time to clearance such as host responses and *Blastocystis *serotypes.

*Blastocystis *infection in these orphans and childcare workers caused no symptoms. This is similar to several studies reporting asymptomatic *Blastocystis *infection in healthy individuals [3,9,28]. It is still unclear why blastocystosis can cause symptoms in some patients. Some specific subtypes of *Blastocystis *sp. such as subtype 3 have been speculated to cause gastrointestinal symptoms; however, no compelling evidence exists for this association [29-32]. It is possible that these orphans and childcare workers were infected by nonpathogenic subtypes. Unfortunately, we have no information of *Blastocystis *subtypes in this study. In addition, it has been shown that immunological status of the infected individual influenced the prevalence of *Blastocystis *infection and its clinical outcomes [33-36]. A few studies identified a significantly higher prevalence of *Blastocystis *infection in an immunocompromised host than in healthy individuals. However, in this orphanage the incidence rate of *Blastocystis *infection in HIV-positive children (IR = 2.3) was not significantly higher than in the HIV-negative group (IR = 1.6). Asymptomatic infection in the HIV-infected group with no higher incidence might be due to their intact immune status. No higher incidence of *Blastocystis *infection may be also the result of strict adherence to universal precautions of the childcare workers in the room for HIV-infected children.

## Conclusions

Our study was the first retrospective cohort study of *Blastocystis *infection conducted in an orphanage with a low incidence of *Blastocystis *infection. Risks factors for *Blastocystis *infection were age, nutritional status and orphans living in the room where their childcare workers were infected. Person-to-person transmission was the most likely to occur either from orphans to childcare workers or from childcare workers to orphans living in the same room. In an institution such as an orphanage, where *Blastocystis *sp. could spread easily, universal precautions such as regular hand washing and careful handling of feces contaminated materials should be taken.

## Competing interests

The authors declare that they have no competing interests.

## Authors' contributions

MM, RR and SL contributed to the conception and design of the study. TN performed stool examination. DP, RR, MM and SL analyzed the data and wrote the manuscript. All authors read and approved the final version that was submitted for publication.
